
JOURNAL INFORMATION
==============================
Journal ID: Wirtschaftsdienst

Wirtschaftsdienst

EISSN: 1613-978X

ARTICLE INFORMATION
==============================
PMCID: PMC9483501
DOI: 10.1007/s10273-022-3277-2
Article ID: 3277
Article version: 1
Subjects: Zeitgespräch

Ausbildungsmarkt unter Druck: die aktuelle Entwicklung und deren Antworten darauf

Training Market Under Pressure: What are the Current Developments and Their Responses?

Lagemann Andreas lagemann@hwwi.org
1
Wedemeier Jan wedemeier@hwwi.org
2
1 grid.435054.3 0000 0001 2227 8589 Hamburgisches WeltWirtschaftsInstitut (HWWI), Oberhafenstraße 1, 20097 Hamburg, Deutschland
2 Hamburgisches WeltWirtschaftsInstitut (HWWI), Fahrenheitstr. 1, 28359 Bremen, Deutschland
Electronic publication date: 2022 Sep 16
Print publication date: 2022
Volume: 102
Issue: 9
First page: 669
Last page: 672
Copyright: © Der/die Autor:in 2022
License: Open Access: Dieser Artikel wird unter der Creative Commons Namensnennung 4.0 International Lizenz veröffentlicht (https://creativecommons.org/licenses/by/4.0/deed.de).
Open Access wird durch die ZBW — Leibniz-Informationszentrum Wirtschaft gefördert.
License URL: https://creativecommons.org/licenses/by/4.0/

Keywords: J21, J6, R1
issue-copyright-statement: © The Author(s) 2022

==============================

Die Zahl der neu abgeschlossenen Ausbildungsverträge ist seit Jahren rückläufig. Der Rückgang lässt sich durch vielfältige Faktoren begründen. Es ist zunächst zu vermuten, dass die konjunkturelle Entwicklung aufgrund der COVID-19-Pandemie und des damit verbundenen Wegfalls von Ausbildungsplätzen einen Erklärungsansatz bietet. Der Trend ist jedoch langfristiger. In einem komplexer werdenden Arbeitsmarkt steigen die Anforderungen an die Auszubildenden und die Unternehmen: Ein zunehmendes Mismatch ist zu beobachten. Auch spielt die demografische Zusammensetzung eine Rolle. Antworten auf den Druck am Ausbildungsmarkt zu finden, ist nicht einfach. Vermeintliche Lösungsansätze erscheinen oft wie ein Ausdruck hilfloser Politik.

Der langfristige Trend auf dem Ausbildungsmarkt ist eindeutig. Die Zahl gemeldeter Bewerbender für einen Ausbildungsplatz ist seit Jahren rückläufig. Während sich deutschlandweit 2009 noch rund 562.000 Nachwuchskräfte für eine Ausbildung bewarben, waren es vor Beginn der Coronapandemie nur noch rund 512.000, 2021 sogar nur noch rund 434.000. Dahinter verbirgt sich zum einem die demografische Entwicklung, zum anderen der steigende Anteil unter jungen Menschen, die einen Hochschulabschluss anstreben. Dagegen ist die Zahl der angebotenen Stellen bis 2019 sogar gestiegen. Das hohe Niveau an Stellen spiegelt die allgemeine positive Entwicklung der Wirtschaft wider. Durch diese Trends lag die Differenz zwischen gemeldeten Stellen (Arbeitsmarktnachfrage) und Bewerbungen (Arbeitsmarktangebot) 2019 bei rund 60.000 und erhöhte sich während der Pandemie auf 78.000 (vgl. Abbildung 1).

Das Missverhältnis zwischen angebotenen Stellen und Bewerbungen geht erwartungsgemäß mit einem Anstieg der unbesetzten Stellen von 17.000 (2009) auf über 63.000 (2021) einher. Auffällig ist jedoch, dass die Zahl der unversorgten Bewerbenden im gleichen Zeitraum ebenfalls gestiegen ist (vgl. Abbildung 2). Für den Rückgang der neu abgeschlossenen Ausbildungsverträge ist also nicht nur eine geringere Zahl an Bewerbenden verantwortlich. Es zeigen sich außerdem Probleme beim Matching zwischen Angebot und Nachfrage (Abraham und Hinz, 2018). In der Theorie können Arbeitsmärkte als „arena for the matching of persons to jobs“ (Sørensen und Kalleberg, 1981, 52) verstanden werden, in der Arbeitnehmerqualifikationen und -erwartungen auf Stellenanforderungen treffen. Wenn diese nicht übereinstimmen, kommt es zu einem Mismatch. Wirft man einen genaueren Blick auf die Daten zum Ausbildungsmarkt, werden drei Formen von Mismatch deutlich (Wieck et al., 2019): ein regionales Mismatch, qualifikatorisches Mismatch und berufsfachliches Mismatch.

## Regionales Mismatch

Betrachtet man die Relation zwischen Bewerbungen und angebotenen Ausbildungsstellen auf Ebene der Landkreise, werden erhebliche regionale Unterschiede deutlich. Vor allem in Baden-Württemberg, Thüringen, Bayern, Mecklenburg-Vorpommern und im Saarland herrscht ein deutlicher Überschuss an Ausbildungsstellen. Im Durchschnitt kommen hier jeweils nur drei Bewerbungen auf vier Stellen. Anders sieht es in Hessen, Nordrhein-Westfalen und Sachsen aus. Hier ist das Verhältnis zwischen Bewerbungen und Stellen im Durchschnitt ausgeglichen. In Berlin gibt es sogar mehr als 140 Bewerbungen pro 100 Stellen (vgl. Abbildung 3). Auch wenn einzelne Regionen eine starke Anziehungskraft ausüben, beschränken sich mehr als 90 % der Suchenden auf einen Radius von 100 km (Christ et al., 2021).

## Qualifikatorisches Mismatch

In vielen Fällen finden Betriebe und Bewerbende trotz passender Ausbildungsstellen nicht zusammen (vgl. Ab-

* Der Artikel basiert auf einem Gutachten „Abschlussbericht der Expertenkommission zur Einführung eines umlagefinanzierten Landesausbildungsfonds in der Freien Hansestadt Bremen" (2022), das zusammen mit Gerhard Bosch, Universität Duisburg-Essen, Hans Dietrich, IAB, Bernhard Nagel, Universität Kassel, Dirk Werner, IW und Clemens Wieland, Bertelsmann Stiftung entstanden ist.

Die Autoren danken Björn Brunke und Lena Subgang, Research Assistants am HWWI, für wertvolle Unterstützung, Hinweise und Recherche.

Abbildung 1
**Gemeldete Berufsausbildungsstellen und gemeldete Bewerbende, jeweils zum 30.9.**

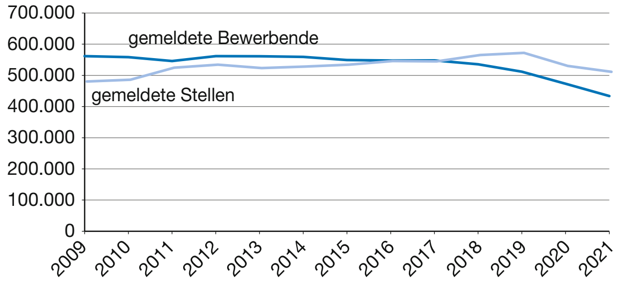

Quellen: BA, 2021a; HWWI.

Abbildung 2
**Unbesetzte Berufsausbildungsstellen und unversorgte Bewerbende, jeweils zum 30.9.**

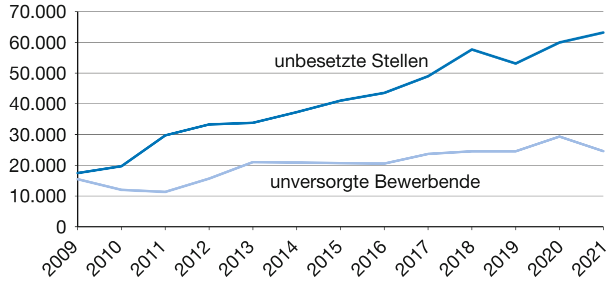

Quellen: BA, 2021a; HWWI.

bildung 2). Dies ist besonders in Großstädten der Fall, da hier überdurchschnittlich viele Bewerbende mit Migrationshintergrund wohnen, die schlechtere Chancen auf eine Ausbildungsstelle haben. Ebenso sind Bewerbende in beliebten Berufen (z. B. Tierpflege, Mediengestaltung, Veranstaltungstechnik) betroffen. In Hinblick auf die soziodemografischen Merkmale unterscheidet sich die Gruppe der Unversorgten vom Durchschnitt aller Bewerbenden. Sie sind öfter über 20 Jahre alt oder sogenannte Altbewerbende, d. h. sie haben in den vergangenen fünf Jahren bereits eine Ausbildungsstelle gesucht. Außerdem verfügen sie häufiger über keinen oder nur einen Hauptschulabschluss (vgl. Tabelle 1). Während dies für rund 30 % der Bewerbenden der Fall ist, fordern fast 50 % der Betriebe mindestens einen Realschulabschluss. Daraus folgt, dass 100 gemeldeten Bewerbenden mit Hauptschulabschluss nur 71 Ausbildungsstellen zur Verfügung stehen. Dieses Ungleichgewicht führt besonders in beliebten Berufen mit hohen Anforderungen (z. B. Informatik, Elektrotechnik) dazu, dass Bewerbende keinen Ausbildungsplatz finden (BA, 2021b). Dazu passt der Befund, dass 49 % der Betriebe, die weniger oder gar nicht mehr ausbilden, die mangelnde Eignung der Bewerbenden als Grund angeben (Eckelt et al., 2020, Wolbers, 2003).

Neben der Bildung spielt der Migrationshintergrund eine entscheidende Rolle bei der Chance, einen Ausbildungsplatz zu finden. Während die Erfolgsquote unter Bewerbenden ohne Migrationshintergrund bei 46 % liegt, ist sie bei Personen mit Migrationshintergrund deutlich geringer (29 %). Auf den ersten Blick liefern die unterschiedlichen soziodemografischen Merkmale dieser beiden Gruppen dafür eine Erklärung. So sind Bewerbende mit Migrationshintergrund durchschnittlich älter, was auf schwierige Übergangswege aus der schulischen in die berufliche Bildung hindeutet. Zudem verfügen Bewerbende mit Migrationshintergrund häufiger über höchstens einen Hauptschulabschluss (37 %) als dies bei denjenigen ohne Migrationshintergrund (28 %) der Fall ist (BIBB, 2021). Doch multivariate Analysen unter Einbeziehung einer Reihe von Kontrollvariablen (z. B. höchster schulischer Abschluss, Durchschnittsnote auf dem Schulabgangszeugnis, etc.) zeigen, dass ein Migrationshintergrund auch bei gleicher Bildung mit schlechteren Chancen auf eine Einmündung in eine betriebliche Ausbildung verbunden ist (Eberhard und Schuß, 2021). Das gilt vor allem für junge Männer mit türkischer bzw. arabischer Herkunft (Beicht und Walden, 2019). Dabei sind Zugewanderte erster Generation, d. h. die selbst eingewandert sind, stärker betroffen als bereits in Deutschland Geborene (Beicht und Walden, 2018).

Abbildung 3
**Bewerbungen pro Ausbildungsstelle nach Landkreisen, Stand 30.9.2021**

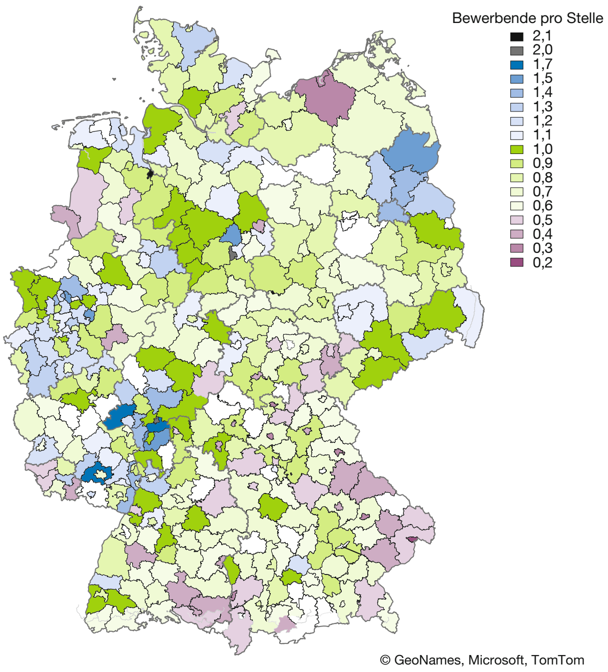

Quellen: BA, 2021a; HWWI.

Tabelle 1

**Bewerbungen nach Merkmalen in Abhängigkeit von deren Vermittlungsstatus**

in %

| | Bewerbungen | Einmündend | Unversorgt |
|---|---|---|---|
| Alter | | | |
| Unter 18 Jahre | 34,1 | 40,6 | 11,3 |
| 18 bis 19 Jahre | 27,5 | 29,1 | 29,2 |
| 20 bis 24 Jahre | 30,4 | 25,9 | 41,8 |
| 25 Jahre und älter | 8,0 | 4,4 | 17,7 |
| Nationalität | | | |
| Deutsche | 81,6 | 85,7 | 77,1 |
| Ausländer:innen | 18,4 | 14,3 | 22,9 |
| Schulabschluss | | | |
| Kein Schulabschluss | 1,6 | 1,5 | 2,2 |
| Mit Hauptschulabschluss | 28,1 | 26,9 | 30,2 |
| Realschulabschluss | 40,1 | 46,2 | 34,7 |
| Fachhochschulreife | 12,9 | 10,5 | 13,3 |
| Allgemeine Hochschulreife | 11,6 | 9,7 | 12,4 |
| Keine Angabe | 5,7 | 5,1 | 7,2 |
| Schulentlassenenjahr | | | |
| Im Berichtsjahr | 52,3 | 56,4 | 42,0 |
| Im Vorjahr | 16,5 | 20,3 | 39,8 |
| In früheren Jahren | 30,4 | 22,8 | 6,5 |
| Keine Angabe | 0,8 | 0,5 | 2,3 |

Quellen: BIBB, 2022; HWWI.

## Berufsfachliches Mismatch

Noch größer als die Zahl der unversorgten Bewerbenden ist die Summe der unbesetzten Stellen, die seit Jahren steigt (vgl. Abbildung 2). Davon sind vor allem Handwerksberufe betroffen. Der Anteil der unbesetzten Ausbildungsstellen an allen gemeldeten Stellen beträgt 16,4 %. Besonders Berufe im Lebensmittelhandwerk (24,0 % unbesetzte Stellen), in der Orthopädie- und Reha-Technik (20,6 %) und im Metallbau (16,8 %) sind betroffen. Aber auch der Bereich Hotel und Gaststätten (25,3 %), Bauberufe (18,0 %) und der Berufskraftverkehr (17,3 %) haben große Probleme, die Stellen zu besetzen. Dagegen zeigt sich für kaufmännische Berufe und in der medizinischen Fachassistenz ein Überangebot an qualifizierten Bewerbenden (BA, 2021b).

Besonders in Berufen, in denen Engpässe an Fachkräften bestehen, ergeben sich Probleme, Ausbildungsstellen zu besetzen (Jansen und Hickmann, 2021). Dabei sind Kleinstbetriebe am stärksten betroffen (Eckelt et al., 2020). Sie konnten 2018 59 % aller angebotenen Stellen nicht besetzen (Klein- und Mittelbetriebe: 37 %; Großbetriebe: 27 %). Kleinstbetriebe gelten unter Bewerbenden dabei als weniger attraktiv, was wiederum dazu führt, dass diesen Unternehmen bei der Ausbildung von Jugendlichen mit schlechteren Schulabschlüssen ein höherer Ausbildungsaufwand entsteht (Severing und Euler, 2018).

## Ausbildungsfondsumlage

Der Handlungsbedarf ist immens, da die Angebots- und Nachfrageschere auseinandergeht und das berufliche, qualifikatorische und regionale Mismatch groß ist. Erschwerend kommt hinzu, dass sich die Probleme am allgemeinen Arbeitsmarkt fortsetzen. Denn 70 % der ausgeschriebenen Stellen für Qualifizierte richten sich an Fachkräfte mit abgeschlossener Berufsausbildung (Jansen und Hickmann, 2021).

Vorschläge wie die Ausbildungsfondsumlage, die aktuell in Berlin und Bremen diskutiert werden, weisen nicht den richtigen Weg. Bei der Ausbildungsfondsumlage geht es vordergründig um ein Anreizinstrument, das die Ausbildungsquote in den Betrieben erhöhen soll. Dahinter steht, dass Unternehmen, die den Nutzen von ausgebildeten Fachkräften erlangen, jedoch vermeiden auszubilden, als Trittbrettfahrende (*free rider*) erachtet werden. Der umlagefinanzierte Ausbildungsfonds zielt auf dieses Trittbrettfahrerverhalten (Form des Marktversagens) ab. Der Ausbildungsfond sieht im Grundsatz vor, dass Unternehmen, die nicht ausbilden, eine Abgabe zahlen. Die volkswirtschaftliche Kosten-Nutzen-Überlegung zur Bereitstellung eines öffentlichen Guts, hier der umlagefinanzierte Landesausbildungsfonds, ist schwierig zu bewerten. Aus Sicht der Gesamtwohlfahrt kann das Gut dann öffentlich bereitgestellt werden, wenn eine effizientere Bereitstellung des Guts möglich ist. Jedoch könnten die Kosten der Einführung und Begleitung eines umlagefinanzierten Ausbildungsfonds den Nutzen des gesamtwirtschaftlichen Wohlfahrtseffekts übersteigen. Die volkswirtschaftlichen Kosten-Nutzen-Relationen einer Einführung sind somit unklar (Fritsch, 2011).

Das Kriterium der Treffsicherheit wird zudem durch das politisch vorgegebene optimale Niveau bestimmt und nicht durch das Angebot der Ausbildungsplätze. Dabei geht die Umlage nicht an das Kernproblem des Matching: Die Ausbildungsfondsumlage ist nur bedingt dazu in der Lage, die Qualifizierung und die Vermittlungsfunktion des Marktes zu übernehmen sowie passgenaue Lösungen anzubieten (Dietrich, 2007). Schwerer wiegt noch, dass die konzeptionelle Idee zum umlagefinanzierten Ausbildungsfonds in einer Zeit entstanden ist, in der ein großes Angebot an Arbeit(skräften) vorgefunden wurde. Demografisch bedingt geht dieses Angebot zurück und zugleich steigt die Nachfrage am Arbeitsmarkt: Das Zukunftsthema ist der Fachkräftemangel am Arbeitsmarkt. Aus dem Mangel heraus werden die Unternehmen nach Lösungen suchen, um Fachkräfte für die Zukunft zu entwickeln und Stellen zu schaffen – auch für Niedrigqualifizierte. Weitere marktwirtschaftliche Eingriffe könnten Fehlanreize, wie strukturkonservierende Wirkungen, setzen.

## Schlussfolgerung

Folgende Lösungsansätze lassen sich exemplarisch benennen und bedürfen weiterer Untersuchungen:

- Die Steigerung der Qualität der schulischen Bildung, insbesondere in den sozialbenachteiligten Stadtteilen bzw. -quartieren, kann die Einmündungen erhöhen. Ein Ansatz ist es, die Schnittstelle zwischen Schule und beruflichem Übergang durch gezielte Schnittstellenförderung und -vermittlung von fehlenden qualifikatorischen Kompetenzen zu verbessern. Die Erhöhung der schulisch-qualifikatorischen Ausbildungsfähigkeit und der laufenden Anpassungen der Kerncurricula an technische Entwicklungen, kann ein Hebel sein, um den Fachkräftemangel (Nachfrage) und Ausbildungsangebot ins (Markt-)Gleichgewicht zu bringen.
- Schulische und zwischenschulische Maßnahmen, wie Vorbereitungswochen im Sinne von Intensivkursen zur dualen Ausbildung, zur Verringerung des qualifikatorischen Mismatch, werden als notwendig erachtet. Daneben bedarf es weitergehender sozialer Begleitung, um die Einmündungsquoten zu erhöhen.
- Es bedarf einer laufenden Bewertung bestehender Instrumente und Ansätze. So äußern beispielsweise einerseits Unternehmen Unterstützungsbedarf bei der Suche nach Bewerbenden und der Qualifizierung von Auszubildenden, anderseits sind den Betrieben viele Instrumente nicht bekannt (Eckelt et al., 2020).
- Da der Anteil der sozialversicherungspflichtig Beschäftigten mit Hochschulabschluss in den vergangenen Jahren enorm an Bedeutung gewonnen hat, könnte die Vergrößerung von Studienangeboten im Dualen System zu einer Verbesserung der Ausbildungsangebote beitragen.
- Aus dem unternehmerischen Dilemma der Fachkräfteanwerbung bedarf es mittelfristig einer (regionalen) Strategie zur Zuwanderung von jungen Erwachsenen für die duale Ausbildung aus EU-Ländern und Drittstaaten, um den zukünftigen Fachkräftebedarf zu deckeln.

Es bedarf der Anwendung eines ökonometrischen Modells zur Abbildung der Gründe des regionalen, qualifikatorischen und berufsfachlichen Mismatches. Vor dem Hintergrund der COVID-19-Pandemie lässt sich über Gründe der aktuellen Ausbildungsmarktmisere nur spekulieren. Eigentlich sollte 2022 ein Erholungsjahr werden, durch die abschwellende Coronapandemie. Allerdings haben sich die Herausforderungen durch den Angriffskrieg Russlands verschärft, die Inflation steigt, die Inputpreise erleben einen sprunghaften Anstieg (Berlemann et al., 2022). Hinzu kommt, dass die Lieferkettenprobleme aus der COVID-19-Krise bis heute anhalten. Die Unsicherheit der Unternehmen ist zurzeit sehr hoch, so auch die Bereitschaft für Neueinstellungen. Die Gründe für den Rückgang in der Pandemie selbst sind durch verschobene Prüfungen, fehlende Berufsorientierung, eingeschränkte Kontaktmöglichkeiten mit potenziellen Bewerbenden und weniger (Schul-)Praktika verursacht. Es bleibt abzuwarten, wie der Ausbildungsmarkt 2023 und in den Folgejahren aussehen wird.

The number of new apprenticeship training contracts has been declining for years. The decline can be explained by a variety of factors. It can initially be assumed that the economic development due to the COVID-19 pandemic and the associated loss of training places offer an explanation. However, the trend is of a longer nature. This article examines proposed solutions, such as the training fund allocation that is currently in discussion, seem more like an expression of futile politics.

Andreas Lagemann ist wissenschaftlicher Mitarbeiter am Hamburgischen WeltWirtschaftsInstitut (HWWI).

Dr. Jan Wedemeier ist dort wissenschaftlicher Mitarbeiter und stellvertretender Leiter des Forschungsbereichs „Räumliche Ökonomik“.


Literatur

Abraham, M. und T. Hinz (2018), Arbeitsmarktsoziologie: Probleme, Theorien, empirische Befunde, 3., überarbeitete und erweiterte Aufl., Springer.
BA (2021a), Statistik der Bundesagentur für Arbeit, Bewerber und Berufsausbildungsstellen, Oktober.
BA (2021b), Statistik der Bundesagentur für Arbeit, Berichte: Arbeitsmarkt kompakt — Situation am Ausbildungsmarkt, Oktober.
Beicht, U. und G. Walden (2018), Übergang nicht studienberechtigter Schulabgänger/-innen mit Migrationshintergrund in vollqualifizierende Ausbildung, BIBB, Report, 6.
Beicht, U. und G. Walden (2019), Der Einfluss von Migrationshintergrund, sozialer Herkunft und Geschlecht auf den Übergang nicht studienberechtigter Schulabgänger/-innen in berufliche Ausbildung, BIBB.
Berlemann M Eurich M Haustein E Inflation in Deutschland gewinnt an Fahrt Wirtschaftsdienst 2022 102 4 319 320 10.1007/s10273-022-3165-9
BIBB (2021), Datenreport zum Berufsbildungsbericht.
BIBB (2022), Datenreport zum Berufsbildungsbericht.
Christ, A., V. Eberhard, M. Heinecke, C. Neuber-Pohl und E. Schuß (2021), Ausbildungsstellensuche in Zeiten der Corona-Pandemie. Belastungen, Einschränkungen und Mehraufwand im Bewerbungsprozess?, BIBB.
Dietrich, H. (2007), Materialsammlung Fachkräftebedarf der Wirtschaft, Institut für Arbeitsmarkt- und Berufsforschung.
Eberhard, V. und E. Schuß (2021), Chancen auf eine betriebliche Ausbildungsstelle von Geflüchteten und Personen mit und ohne Migrationshintergrund, BIBB.
Eckelt, M., S. Mohr, C. Gerhards und C. Burkard (2020), Rückgang der betrieblichen Ausbildungsbeteiligung: Gründe und Unterstützungsmaßnahmen mit Fokus auf Kleinstbetriebe, BIBB.
Fritsch, M. (2011), Marktversagen und Wirtschaftspolitik, Vahlen.
Jansen, A.und H. Hickmann (2021), Lockdown am Ausbildungsmarkt: Folgen für die Fachkräftesicherung, KOFA-Studie, 3.
Severing, E. und D. Euler (2018), Ausweitung der Ausbildungsressourcen, Bertelsmann Stiftung.
Sørensen, A. B. und A. L. Kalleberg (1981), An Outline for a Theory of the Matching of Persons to Jobs, Academic Press, 49–74.
Wieck, M., S. Seeber, V. Baethge-Kinsky, P. Geiser, R. Busse, C. Michaelis und V. Boschke (2019), Ländermonitor berufliche Bildung 2019: Ein Vergleich der Bundesländer mit vertiefender Analyse zu Passungsproblemen im dualen System (1. Aufl.), wbv Publikation.
Wolbers M H J Job Mismatches and their Labour- Market Effects among School-Leavers in Europe European Sociological Review 2003 19 3 249 266 10.1093/esr/19.3.249
